# Comprehensive assessment of lower limb edema and its association with quality of life among men with prostate cancer

**DOI:** 10.1007/s00520-025-09613-4

**Published:** 2025-06-16

**Authors:** Sandra Jensen, Andreas Røder, Sandi Hayes, Gitte Sone Larsen, Mikkel Fode, Peter Busch Østergren, Muhammad Munther Nasir Al-Hamadani, Christoffer Johansen, Bolette Skjødt Rafn

**Affiliations:** 1 Cancer Survivorship and Treatment Late Effects, Department of Oncology, University Hospital of Copenhagen, Rigshospitalet, Copenhagen, Denmark; 2 Urological Research Unit, Department of Urology, Faculty of Health and Medical Sciences, University of Copenhagen, Rigshospitalet, Copenhagen, Denmark; 3 Department of Clinical Medicine, University of Copenhagen, Copenhagen, Denmark; 4 Cancer Council Queensland, Queensland, Australia; 5 University of Queensland, Brisbane, Australia; 6 Griffith University, Brisbane, Australia; 7 Department of Urology, Copenhagen University Hospital, Herlev and Gentofte Hospital, Herlev, Denmark

**Keywords:** Lymphedema, Objective measurement, Health-related quality of life, Bioimpedance spectroscopy, Prostate cancer

## Abstract

**Purpose:**

Lower limb edema (LLE) is characterized by swelling due to fluid accumulation and is an under-recognized condition in men with prostate cancer. This study investigated the prevalence of LLE and explored its impact on daily living, depression, and health-related quality of life (HRQoL).

**Methods:**

This cross-sectional study included men with prostate cancer who attended follow-up at the Department of Urology, Rigshospitalet, Denmark, during a 3-month period. LLE was defined as an L-Dex ≥ 10, measured by using bioimpedance spectroscopy, combined with self-reported symptoms (≥ 2 of heaviness, swelling, or tightness) using items from European Organisation for Research and Treatment of Cancer Quality of Life (EORTC) QLQ-VU34. HRQoL, depression, and the impact of LLE on daily living were assessed using self-reported questionnaires.

**Results:**

Among 401 patients, LLE was identified in 45 (11%) patients. Self-reported swelling before diagnosis, comorbidities, BMI ≥ 30, and androgen deprivation therapy (ADT) showed the highest odds of LLE. Patients with LLE demonstrated lower HRQoL scores in global health, physical, role, and social functioning and reported higher bowel and hormonal treatment-related symptoms (*p* < 0.05). LLE impacted daily activities, including walking (42%) and clothing choices (41%). Most patients (76%) wanted more information on managing LLE symptoms.

**Conclusions:**

LLE is prevalent among men with prostate cancer and is associated with poorer HRQoL and daily living. Despite its impact, many patients report a lack of information and express a desire for more knowledge about the condition. Improved patient education and attention to potential underlying causes are crucial for timely treatment of LLE.

**Supplementary Information:**

The online version contains supplementary material available at 10.1007/s00520-025-09613-4.

## Introduction

Prostate cancer and its treatment can contribute to lower limb edema (LLE) [1]. LLE is an accumulation of excess fluid within the body’s tissues, commonly presenting as swelling in areas such as the legs, ankles, and feet [2]. LLE can arise from multiple factors, including cancer itself, cancer treatments, and other systemic health disorders [3, 4]. This condition typically presents with swelling, as well as sensations of heaviness and tightness in the limbs and genital area. While initially reversible, LLE can progress to a chronic condition over time, with permanent intradermal fibrosis and tissue inflammation [3, 4].

The risk of LLE for men with prostate cancer is explained through several mechanisms. Prostate cancer often spreads to lymph nodes in the pelvic area, and this lymphatic obstruction may cause lymphedema in the legs and genital area [4]. In addition, treatment-related factors may play a substantial role in the development of LLE. Hormone therapies for prostate cancer, such as abiraterone, GnRH agonists, GnRH antagonists, and enzalutamide, are associated with increased fluid retention or elevated blood pressure, which may lead to LLE [5–7]. Also, chemotherapy, e.g., docetaxel can contribute to LLE by increasing blood vessel permeability, leading to fluid leakage into the surrounding tissue, and disrupting fluid balance and lymphatic drainage [8–10]. Finally, surgery and radiation treatments targeting pelvic lymph nodes, while important for prognosis, may damage the lymphatic system, thereby exacerbating the risk of LLE [1].

Aside from the tumor topography and the treatment of prostate cancer, general health conditions that become more common by age, such as hypertension, high body mass index (BMI), chronic venous insufficiency, and heart failure, are also significant factors, which contribute as etiological agents for LLE. Together, these conditions increase the risk of LLE [11–14]. Men with prostate cancer are however often inadequately informed about LLE as a potential late effect of cancer and the treatment thereof [15].

LLE can significantly impact quality of life, causing physical discomfort, psychological distress, and increased depressive and somatic symptoms [16, 17]. Despite these burdens, studies investigating LLE in men with prostate cancer are limited. Previous research has often focused on small cohorts or populations of women, with findings that may not be directly applicable to men [18–20]. Additionally, many studies have relied on inconsistent definitions and non-standardized assessment methods, leading to varying prevalence estimates from 0 to 9% after radiation therapy alone, 0–14% following radical prostatectomy with pelvic lymph node dissection (PLND), to 18–29% when both treatments are combined [1].

This study addresses these gaps by combining patient-reported outcomes and objective measurements, to provide a comprehensive assessment of LLE and to investigate its prevalence and impact on activities of daily living, depression, and health-related quality of life among men with prostate cancer.

## Materials and methods

### Design and participants

This cross-sectional study included men with prostate cancer attending follow-up visits at the Department of Urology, Rigshospitalet, Denmark, between November 1 st, 2022, and January 31 st, 2023. Inclusion criteria were a diagnosis of prostate cancer, age ≥ 18 years, ability to read and understand Danish to complete patient-reported outcomes. Patients were eligible if they had received any treatment modality for prostate cancer, both active (i.e., surgery or androgen deprivation therapy (ADT)) or passive (i.e., watchful waiting or active surveillance). Patients with pacemakers were excluded, as this is a contraindication for the measurement of LLE by bioimpedance spectroscopy (BIS). All patients were contacted by telephone and invited to one in-person visit to complete patient-reported outcomes and undergo measurements with BIS. Two attempts to reach each patient by telephone was conducted to offer participation.

### Outcome measures

#### Socio-demographic and clinical characteristics

Socio-demographic characteristics and general comorbidities were self-reported. Clinical and treatment-related variables were extracted from the medical records. The Gleason score was obtained from prostatectomy specimens for patients who underwent surgery, and from biopsies for others. Body weight was measured at the in-person visit. All data were registered in the REDCap database [21].

#### Measurement of LLE

Each participant was assessed twice using the BIS device (SOZO® Digital Health Platform by ImpediMed), and the average lymphedema index (L-Dex) was recorded. The L-Dex score is calculated from the impedance ratio between limbs and provides a measure of extracellular fluid levels, with values of ≥ 10 indicating the presence of lymphedema [22]. Using the vulvar cancer-specific subscale of the European Organization for Research and Treatment of Cancer Quality of Life Questionnaire (EORTC QLQ–VU34 items 39, 40, 41, 44, 45, and 46), participants were asked to rate presence of heaviness, swelling, or tightness in the legs, groin, or genital area within the past 2 weeks using a 4-point Likert scale, with response options of 1 = “not at all,” 2 = “a little,” 3 = “quite a bit,” and 4 = “very much” [23]. Swelling, tightness, and heaviness are commonly used to assess lymphedema-related symptoms; however, these aspects are not sufficiently covered in prostate cancer-specific tools, which is why the EORTC QLQ-VU34 items were chosen [24]. The presence of LLE was defined as L-Dex of ≥ 10 [25, 26] and at least one self-reported LLE-associated symptom (heaviness, swelling or tightness) of ≥ 2.

### Patient-reported outcomes

#### Health-related quality of life

Health-related quality of life (HRQoL) was measured by EORTC QLQ-C30 which includes functional scales (physical, role, cognitive, emotional, and social functioning), symptom scales (fatigue, pain, and nausea/vomiting), a global health status/QoL scale, and single symptom items. Functional and symptom scales are rated on a 4-point Likert scale (1 = “not at all” to 4 = “very much”), while the global health status/QoL scale uses a 7-point scale (1 = “very poor” to 7 = “excellent”). Higher scores on the functional and global health scales indicate better functioning and HRQoL, whereas higher scores on symptom scales reflect greater severity [27, 28].

#### Prostate cancer-specific HRQoL

The EORTC QLQ-PR25 is a prostate cancer-specific subscale that includes symptom and functional domains: urinary symptoms, bowel symptoms, hormonal treatment-related symptoms, and sexual functioning. Higher scores on symptom domains indicate more severe symptoms, and higher scores on the sexual functioning domain represent better sexual functioning [29].

#### Depression

Depression was assessed using the Major Depression Inventory (MDI), which cover the frequency of 10 depressive symptoms within the last 2 weeks, each rated on a 6-point Likert scale (0 = “at no time” to 5 = “all the time”). The total score ranges from 0 to 50, with scores of ≤ 20 indicating no depression, 21–25 mild depression, 26–30 moderate depression, and ≥ 31 severe depression [30, 31].

#### Impact of LLE

Impact of LLE was administered to participants who reported LLE-associated symptom (heaviness, swelling or tightness) of ≥ 2 using The Lymphoedema Genito-Urinary Cancer Questionnaire (LGUCQ) tool, which accesses the impact on daily living. Items are rated on a Likert scale from 0 to 3, with 0 indicating “not at all” and 3 indicating “very much” [32].

### Ethics

The Capital Region of Copenhagen Ethical Committee judged that ethical approval was not required (F-23004790) and The Danish Data Protection Agency (R-22071980) approved contacting patients by phone for participation. All patients were informed verbally and in writing about the study. Only men who provided written informed consent were included. The Helsinki Declaration was followed for all aspects of the study.

### Statistical analysis

Descriptive statistics were used to summarize socio-demographic and clinicopathological characteristics, with categorical variables presented as frequencies, and continuous variables as mean ± standard deviation or median and 25 th percentile (Q1), 75 th percentile (Q3), depending on distribution. Welch’s *t*-test, *χ*^2^ tests, or Mann–Whitney *U* test was used to compare clinicopathological variables and depression between participants with and without LLE. LLE prevalence and severity of symptoms were presented descriptively, and LLE treatment variables were analyzed as binary outcomes. Stepwise backward regression using Akaike Information Criterion was applied to identify variables explaining LLE likelihood, followed by multivariate logistic regression to calculate odds ratios (ORs) with 95% confidence intervals (CIs) for the final model predictors. Differences in HRQoL were analyzed using Welch’s *t*-test and multiple linear regression, with results presented as mean differences with 95% CI across three models: (a) unadjusted; (b) adjusted for age, BMI, comorbidities associated with LLE, self-reported comorbidities, and pre-diagnosis swelling; and (c) further adjusted for time since diagnosis and treatment modalities, including radical prostatectomy, chemotherapy, radiation therapy, ADT, and lymph node removal. Participants and non-participants were compared on age using Welch’s *t*-test, as age was the only variable available for the non-participants.

## Results

**Fig. 1:**
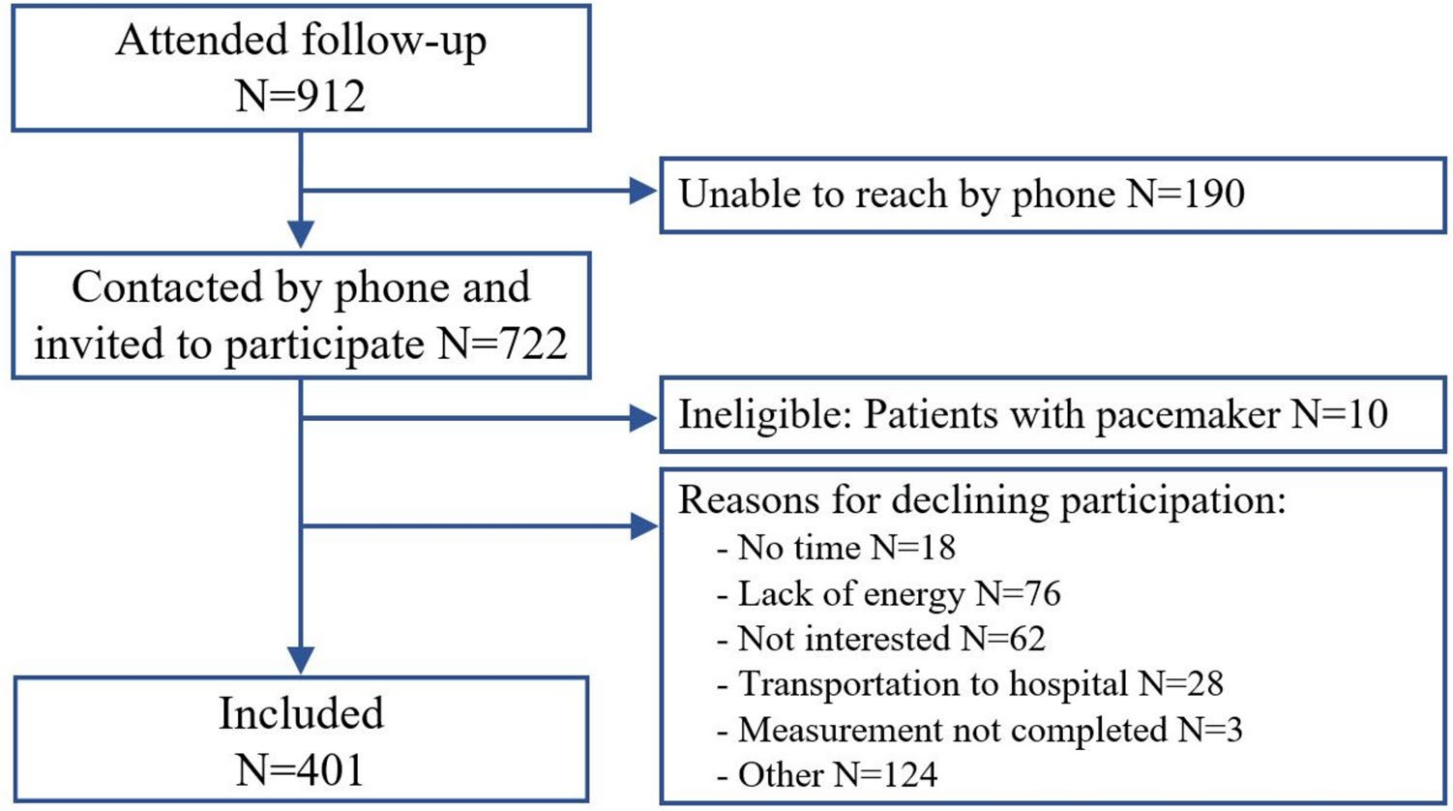
Flowchart

A total of 912 patients were scheduled for follow-up visits for prostate cancer at Rigshospitalet between November 2022 and January 2023. Of these, 722 patients could be reached by telephone and invited to the study. Subsequently, 401 (56%) agreed to participate in the study and completed both patient-reported outcomes and BIS measurements. Ten patients were excluded due to having a pacemaker and 311 patients declined participation. Most patients declined due to lack of energy, no interest, or other reasons (Fig. 1). Patients who declined to participate in the study were older than those who participated (74 years ± 9 vs. 72 years ± 8, *p* < 0.001).

The majority of participants were either overweight or obese (*n* = 265, 66% had BMI ≥ 25), were either former or current smokers (*n* = 224, 58%), and almost one-third lived alone (*n* = 108, 27%) (Table 1). On average, participants were 5 years (Q1: 2, Q3: 8) post prostate cancer diagnosis. Half of the participants had undergone radical prostatectomy (*n* = 203, 51%) with PLND in 36% of the cases (*n* = 146). On average 17 ± 5 nodes were removed, and 2 (Q1: 1, Q3: 3) showed metastases. Approximately one in two (*n* = 217, 54%) had or were receiving ADT, while 27% (*n* = 107) had received radiation therapy, and 15% (*n* = 59) were in watchful waiting or active surveillance.

**Table 1:** Participant characteristics

	**All** ***N* = 401**	**LLE** ***N* = 45**	**No LLE** ***N* = 356**	**Difference**	***p*-value**
	Mean ± SD Median (Q1, Q3) *N* (%)	Mean ± SD Median (Q1, Q3) *N* (%)	Mean ± SD Median (Q1, Q3) *N* (%)	Mean or median difference (95% CI)	
**Age**	72 ± 8	75 ± 7	71 ± 8	4 (2, 6)	0.002
**BMI, kg/m**^**2**^					< 0.001
**< 25** **25–29.9** **≥ 30**	136 (34%) 182 (45%) 83 (21%)	9 (20%) 17 (38%) 19 (42%)	127 (36%) 165 (46%) 64 (18%)		
**Highest level of education**					0.908
**Primary or secondary school** **Vocational education** **Continued education (2–3 years)** **Continued education (3–4 years)** **Continued education (> 4 years)** **Other**	36 (9%) 102 (25%) 37 (9%) 85 (21%) 126 (31%) 15 (4%)	4 (9%) 9 (20%) 3 (7%) 11 (24%) 16 (36%) 2 (4%)	32 (9%) 93 (26%) 34 (10%) 74 (21%) 110 (31%) 13 (4%)		
**Annual household income, DKK**					0.611
**100.000–250.000** **250.000–400.000** **400.000–700.000** ** > 700.000**	91 (23%) 101 (25%) 108 (27%) 101 (25%)	13 (29%) 11 (24%) 9 (20%) 12 (27%)	78 (22%) 90 (2%) 99 (28%) 89 (25%)		
**Living alone**	108 (27%)	10 (22%)	98 (28%)		0.564
**Smoking**					0.515
**Yes** **No** **Former smoker**	55 (14%) 160 (42%) 169 (44%)	6 (13%) 19 (42%) 20 (44%)	49 (14%) 141 (40%) 149 (42%)		
**Years since diagnosis**	5 (2, 8)	6 (3, 10)	5 (2, 8)	1 (0, 2)	0.149
**PSA score**	10 (7, 18)	12 (8, 15)	10 (7, 18)	1 (− 1, 3)	0.155
**Gleason score**					0.179
**6** **7 (3 + 4)** **7 (4 + 3)** **8** **9–10**	65 (16%) 131 (33%) 78 (20%) 33 (8%) 90 (24%)	5 (12%) 13 (30%) 5 (12%) 6 (14%) 14 (33%)	60 (17%) 118 (33%) 73 (21%) 27 (8%) 76 (22%)		
**Stage**					0.293
**I** **II** **III** **IV**	40 (10%) 140 (35%) 116 (28%) 105 (26%)	6 (13%) 10 (22%) 15 (33%) 14 (31%)	34 (10%) 130 (37%) 101 (28%) 91 (26%)		
**Reported swelling before diagnosis**					0.002
**No swelling** **Swelling** Missing*	223 (56%) 52 (13%) 136 (34%)	19 (63%) 11(37%) 15 (33%)	204 (87%) 31 (13%) 121 (34%)		
**Self-reported comorbidities**					0.027
**No comorbidities** **1–2 comorbidities** **3–5 comorbidities**	203 (51%) 166 (41%) 32 (8%)	18 (40%) 19 (42%) 8 (18%)	185 (52%) 147 (41%) 24 (7%)		
**Comorbidities related to edema**					
**Heart failure** **Hypertension** **Valvular heart disease** **Venous thrombosis**	17 (4%) 212 (53%) 76 (19%) 21 (5%)	6 (13%) 36 (80%) 16 (36%) 7 (16%)	11 (3%) 176 (49%) 60 (17%) 14 (4%)		0.005 < 0.001 0.005 0.029
**Radical prostatectomy**	203 (51%)	15 (33%)	188 (53%)		0.021
**Lymph node removal**	146 (36%)	16 (36%)	130 (37%)		1.000
**No. of lymph nodes removed**	17 ± 5	18 ± 11	17 ± 8	1 (−6, 8)	0.758
**No. of lymph node metastases**	2 (1, 3)	1 (1,2)	2 (1, 3)	1 (0, 1)	0.351
**ADT**	217 (54%)	32 (71%)	185 (52%)		0.023
**Radiation therapy**	107 (27%)	12 (27%)	95 (27%)		1.000
**Chemotherapy treatment**	69 (17%)	9 (20%)	60 (17%)		1.000
**WW or AS**	59 (15%)	8 (14%)	51 (14%)		0.695

*N* number, *SD* standard deviation, *Q1* 25 th percentile, *Q3* 75 th percentile, *95% CI* 95% confidence interval, *BMI* body mass index, *Annual income* annual income before tax in DKK, *ADT* androgen deprivation therapy, *WW* watchful waiting, *AS* active surveillance. *This question was introduced after the study had commenced, which explains the substantial amount of missing data

### LLE

LLE was identified in 11% (*n* = 45) of participants (Supplementary file 1). Of these, 34 (76%) had unilateral LLE, while 11 (24%) had bilateral LLE.

Presence of LLE was significantly associated with older age (mean difference of 4 years; 95% CI: 2 to 6), obesity (BMI ≥ 30; 42% vs. 18%), and a history of self-reported swelling at pre-diagnosis (37% vs. 13%) (Table 1). Additionally, participants with LLE were less likely to undergo radical prostatectomy (33% vs. 53%), more likely to receive or had received ADT (71% vs. 52%), reported more often to have 3–5 comorbidities (18% vs. 7%). Comorbidities like heart failure, valvular disease, hypertension, and venous thrombosis were also more common in participants with LLE (*p* < 0.05).

The variables most strongly associated with LLE were identified using stepwise backward regression based on Akaike Information Criterion. Multivariate logistic regression identified that “quite a bit” or “very much” swelling before diagnosis had the strongest association with LLE (OR: 47, 95% CI: 7 to 453). Higher odds were also observed for heart failure (OR: 16, 95% CI: 3 to 87), self-reported metabolic diseases (OR: 16, 95% CI: 1 to 202), BMI ≥ 30 (OR: 7, 95% CI: 2 to 28), and having received ADT (OR 4, 95% CI: 1 to 12) No significant associations were found for age, radiotherapy, COPD or chronic bronchitis, or valvular heart disease (Supplementary file 2 and Table 2). Further, having > 20 lymph nodes removed was not a significant predictor (OR: 1.47, 95% CI: 0.28 to 6.14).

**Table 2:** Variables that most strongly explain LLE

	**OR**	**95% CI**	***p*-value**
**Age**	1.1	1.0, 1.1	0.080
**BMI kg/m**^**2**^			
< 25	-		
25–29.9	0.6	0.2, 2.2	0.4
≥ 30	7.1	2.1, 27.8	0.003
**Radiation therapy**			
No	-	-	
Yes	0.3	0.1, 1.0	0.053
**Swelling before diagnosis**			
Not at all	-	-	
A little	1.00	0.3, 3.4	> 0.9
Quite a bit or very much	47.3	7.3, 453	< 0.001
**ADT**			
No	-	-	
Yes	3.6	1.2, 11.5	0.023
**Metabolic diseases**			
No	-		
Yes	16.1	1.3, 202	0.025
**COPD or chronic bronchitis**			
No	-	-	
Yes	0.1	0.00, 1.3	0.092
**Heart failure**			
No	-	-	
Yes	16.1	3.3, 86.5	< 0.001
**Valvular heart disease**			
No	-	-	
Yes	2.4	0.9, 6.5	0.079

*OR* odds ratio, *CI* 95% confidence interval, *BMI* body mass index, *ADT* androgen deprivation therapy, *COPD* chronic obstructive pulmonary disease. Variables included in this multivariate logistic regression were selected through stepwise backward regression as those most strongly associated with the likelihood of LLE (step 15), see supplementary file 2

### Prevalence of LLE based on specific treatments

When examining the specific treatment combinations, it was observed that the prevalence of LLE was highest among patients whose only treatment was ADT (*n* = 10, 26%), ADT with radical prostatectomy with PLND (*n* = 3, 27%), ADT, radical prostatectomy with PLND, radiation therapy, and chemotherapy (*N* = 7, 28%) (Supplementary file Cite ESM.3). By contrast, 14% (*n* = 8) of participants who received watchful waiting or active surveillance had LLE. Notably, these individuals were also obese (*n* = 7) and/or had hypertension (*n* = 6) which can lead to LLE.

### Relationship between LLE and HRQoL and depression

Patients with LLE were more likely to report lower scores in several HRQoL domains compared to those without LLE (Table 3), specifically in Global Health Status/QoL by − 12 (95% CI: − 19 to − 6), Physical Functioning − 13 (95% CI: − 20 to − 6), Role Functioning − 19 (95% CI: − 29 to − 9), and Social Functioning − 13 (95% CI: − 22 to − 4). Additionally, patients with LLE were more likely to report more severe bowel symptoms and hormonal treatment-related symptoms. These differences remained statistically significant after adjusting confounders. There was no difference in depression between the groups (Supplementary file 4).

**Table 3:** Relationship between lower limb edema and health-related quality of life

	**Crude**	***p*-value**	**Adjusted model 1**	***p*-value**	**Adjusted model 2**	***p*-value**
	**Difference of means (95% CI)**		**Difference of means (95% CI)**		**Difference of means (95% CI)**	
**EORTC-QLQ-C30**						
**Global health status/QoL**	− 12 (− 19, − 6)	< 0.001	− 10 (− 18, − 2)	0.012	− 10 (− 18, − 2)	0.018
**Physical functioning**	− 13 (− 20, − 6)	< 0.001	− 10 (− 17, − 4)	0.007	− 10 (− 17, − 4)	0.001
**Role functioning**	− 19 (− 29, − 9)	< 0.001	− 13 (− 22, − 4)	0.001	− 12 (− 22, − 3)	0.011
**Social functioning**	− 13 (− 22, − 3.6)	< 0.001	− 14 (− 22, − 5)	0.002	− 13 (− 22, − 4)	0.003
**Cognitive functioning**	− 8 (− 15, − 1)	0.034	− 7 (− 14, 1)	0.072	− 6 (− 13, 1)	0.102
**Emotional functioning**	− 4 (− 11, 12)	0.165	− 7 (− 13, − 1)	0.027	− 6 (− 13, 0)	0.041
**EORTC-QLQ-PR25**						
**Urinary symptoms**	10 (3, 16)	0.006	4 (− 3, 11)	0.264	3 (− 4, 11)	0.374
**Incontinence**	5 (− 7, 17)	0.396	9 (− 2, 20)	0.112	12 (0, 23)	0.056
**Bowel symptoms**	6 (1, 12)	0.018	6 (2, 11)	0.009	7 (3, 12)	0.002
**Hormonal treatment-related symptoms**	10 (5, 15)	< 0.001	8 (3, 14)	0.005	7 (2, 13)	0.010
**Sexual activity**	− 6 (− 14, 3)	0.190	2 (− 10, 13)	0.795	4 (− 7, 15)	0.501
**Sexual functioning**	2 (− 8, 11)	0.682	6 (− 6, 19)	0.314	7 (− 6, 20)	0.274

*95% CI* 95% confidence interval

Adjusted Model I = linear regression adjusted for, age, body mass index (BMI), comorbidities associated with development of edema, self-reported comorbidities and swelling before diagnosis

Adjusted Model II = linear regression adjusted for Adjusted model I + time since diagnosis, treatment (radical prostatectomy, chemotherapy, radiation therapy, androgen deprivation therapy (ADT), lymph node removal)

### Impact of LLE on daily living

**Fig. 2:**
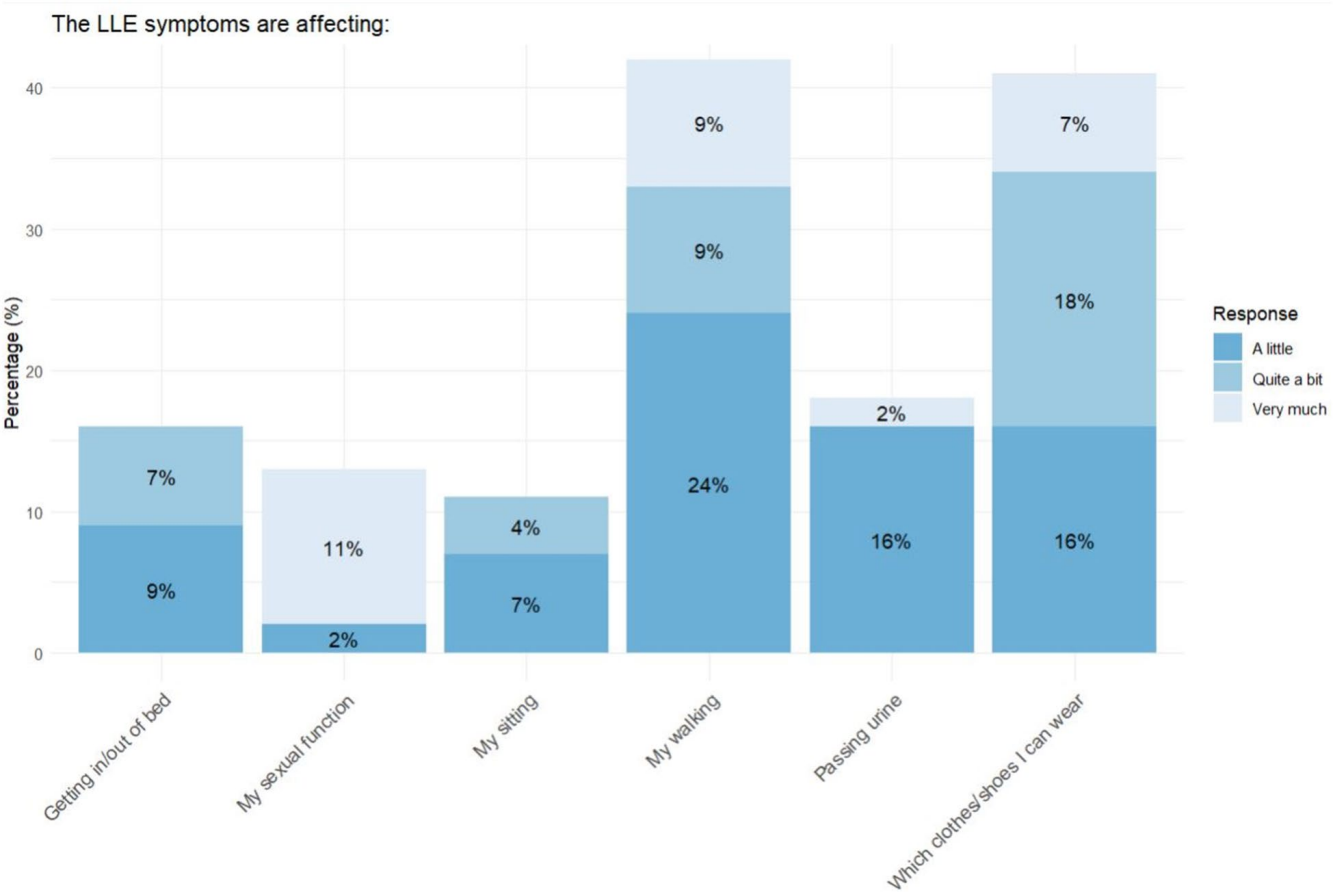
The LLE symptoms are affecting

Of the 45 participants with LLE many reported that the LLE symptoms impacted essential daily activities (Fig. 2 and Supplementary file 5). This included their ability to get in and out of bed (*n* = 7, 16%), their sexual function (*n* = 6, 13%), sitting (*n* = 5, 11%), walking (*n* = 19, 42%), passing urine (*n* = 8, 18%), and choosing appropriate clothing or footwear (*n* = 18, 41%). Sixteen (36%) reported experiencing two or more of these challenges. Additionally, 9% (*n* = 4) of these participants required antibiotics for leg infections. Despite these challenges, 35 (78%) participants had not received advice or treatment for managing LLE symptoms, and 34 (76%) expressed a desire for more information on LLE.

## Discussion

This study represents the first investigation of the prevalence of LLE and its association with HRQoL, depression, and daily living among men with prostate cancer, using both BIS and patient-reported outcomes. We found an overall prevalence of 11% across various treatment modalities and stages of the disease. LLE was associated with lower HRQoL, increased treatment-related symptoms, and impairments to daily activities. However, no association between LLE and depression was observed. Moreover, presence of LLE was significantly associated with older age, higher BMI, more comorbidities, self-reported swelling in the lower limbs prior to diagnosis, and increased exposure to ADT.

Our findings align with results from other studies. A prospective observational study defining edema as self-reported swelling in the legs or ankles found that high BMI (> 30), a higher Charlson Comorbidity Index, and ADT significantly increased LLE risk after radiation therapy in 302 men with prostate cancer. However, only high BMI remaining significant in multivariate analysis [33]. Similarly, in our study, obesity was associated with a higher prevalence of LLE, with 80% of affected men classified as overweight or obese and 71% having ADT exposure. ADT can induce metabolic changes, including weight gain, which may further contribute to LLE risk [34, 35]. These findings underscore the importance of identifying potential underlying causes of LLE, including metabolic changes and weight gain, for which targeted interventions like exercise and diet programs are effective [36]. Moreover, we found that patients with LLE were less likely to undergo radical prostatectomy. Patients undergoing surgery are often in better overall health, while those with, e.g., metabolic disorders or comorbidities face higher surgical risks [37]. In our study, patients with LLE had more comorbidities, possibly explaining their ineligibility for surgery. However, further research is needed to confirm this.

When comparing prevalence for LLE across treatment modalities, substantial variability emerges. The highest prevalence in our study (26%) was observed among patients receiving ADT, a treatment typically reserved for older individuals with advanced or metastatic disease, who are at greater risk of developing edema [38]. A prospective observational study found a prevalence of 23% for self-reported moderate to severe lymphedema symptoms at a median of 15 months post-treatment among men receiving radiotherapy and ADT [33]. In comparison, we found a prevalence of LLE of 13% among patients who underwent radiotherapy and ADT.

Among a small subset of patients undergoing radical prostatectomy with PLND, we found a prevalence of LLE of 4%, which aligns with prevalence of 0–14% reported by a 2022 systematic review of 18 studies and 9223 men [1]. However, for patients treated with multiple therapies, including radical prostatectomy with PLND, radiation therapy, and ADT, the prevalence of LLE in our study was also 4%. This is substantially lower than the 18–29% prevalence reported in the systematic review for cases combining prostatectomy with radiotherapy [1]. However, the findings from our study are based on a small subset of patients receiving these treatments, and differences in methods for assessing may explain the difference. Notably, the systematic review highlights a significant limitation, as most included studies lacked definitions or description of how lymphedema was identified, with only one study using a standardized questionnaire for patient-reported symptoms [1]. In contrast, a cross-sectional study used both circumferential limb measurements and International Society of Lymphology staging criteria to define LLE among men treated with postoperative radiotherapy following radical prostatectomy ± PLND [39] and reported a prevalence of 13.9%. Interestingly, the study identified diabetes mellitus, low physical activity, and shorter time between surgery and radiotherapy as significant predictors of LLE [39]. While our study did not collect data on exercise habits or time between treatments, we similarly found strong associations between LLE and metabolic comorbidities such as heart failure and obesity. Moreover, both studies observed that the number of lymph nodes removed was not a significant predictor in multivariate models, suggesting that comorbidity burden and systemic effects of treatment may be more important than surgical extent alone.

These discrepancies illustrate the challenges of comparing LLE rates across studies, as variations in treatment regimens, disease stages, and assessment methods can all influence prevalence estimates. While patient-reported outcomes, clinical evaluations, and objective measurements provide valuable insights, they will yield varied results depending on the criteria and tools used.

Our analysis revealed that the presence of LLE was associated with lower HRQoL. Similarly, a survey-based study including 54 men with lymphedema following radical prostatectomy with PLND found that men with persistent lymphedema experienced significantly greater burdens in daily living compared to men whose lymphedema had resolved [17]. Likewise, a case–control study from Portugal including 12 men and 68 women found that individuals with lymphedema exhibited significantly higher levels of depressive symptoms, compared to controls, highlighting its psychological burden [16]. In contrast, our study found no difference in depression scores between patients with and without LLE. This discrepancy may be explained by the often higher prevalence of depression among women, as suggested by other studies [40]. However, these results highlight the relationship between LLE and physical and emotional burden, emphasizing the need for patient support.

Notwithstanding the recognized burden of LLE, our study revealed that 78% of participants with LLE had not received advice or treatment for managing their symptoms, and 76% expressed a need for more information. While we cannot ascertain whether participants received advice on managing underlying factors, such as lifestyle interventions to reduce the risk of metabolic syndrome, it remains crucial to address potential underlying causes of LLE to enable targeted and effective treatment. However, our findings indicate a substantial gap in patient education, leaving many to cope with the challenges of LLE without adequate guidance. Similarly, other studies have identified a lack of structured support for LLE management, where the condition is often described as the “forgotten vascular disease” or labeled as “under-recognized and under-treated” [1, 15, 41, 42]. This trend highlights a need for greater awareness, management, and support for LLE for men with prostate cancer.

In this study, we defined LLE prevalence as the presence of both self-reported symptoms and an L-Dex score > 10 using BIS. BIS is a widely used tool for detecting lymphedema, particularly in breast cancer care [43]. The L-Dex threshold of > 10 indicates LLE which is based on the normal distribution of L-Dex in a healthy population (mean + 3 SD) [44]. By including self-reported symptoms alongside objective measure, we ensured that the swelling was both clinically detectable and noticeable to participants. If we had relied solely on self-reported symptoms, the prevalence would have ranged from 17% to 46% potentially overestimating true prevalence, as swelling perception may be influenced by factors beyond physical changes in LLE [45]. While self-reported symptoms are moderately reliable, prior studies indicate that they are best complemented by highly reliable measurements like BIS [45]. Therefore, combining self-reports with BIS enhances overall accuracy and comprehensiveness of LLE assessment.

### Strengths and limitations

The most important strength of this study is the large sample who underwent both objective and subjective assessments for a comprehensive evaluation of LLE. Yet, a key limitation is its cross-sectional design, which limits causal inference. Additionally, the absence of a control group without prostate cancer restricts differentiation between treatment-related and population-level risk factors. Although the analyses were adjusted for several variables, some confounders may not have been accounted for.

## Conclusion

Among 401 men with prostate cancer, we found a prevalence of 11% for LLE. LLE was associated with lower HRQoL and impairment in daily living. Moreover, presence of LLE was significantly associated with older age, higher BMI, more comorbidities, self-reported swelling prior to diagnosis, and increased ADT exposure. Most participants expressed a desire for more information on how to manage their LLE symptoms. These findings highlight the importance of raising awareness, addressing treatable causes and providing support to improve HRQoL and functional outcomes. Well-designed prospective studies with baseline measurements before cancer treatment are needed to clarify how specific treatments affect LLE and to identify those at greatest risk.

## Supplementary Information

Below is the link to the electronic supplementary material.

ESM 1 (JPG 53.3 KB)

ESM 2 (DOCX 23.9 KB)

ESM 3 (DOCX 14.8 KB)

ESM 4 (DOCX 13.7 KB)

ESM 5 (DOCX 15.5 KB)

## Data Availability

Data is available upon request to the corresponding author.

## References

[1] Clinckaert A, Callens K, Cooreman A, Bijnens A, Moris L, Calster CV et al (2022N 18) The prevalence of lower limb and genital lymphedema after prostate cancer treatment: a systematic review. Cancers 14(22):5667 36428759 10.3390/cancers14225667 PMC9688147

[2] Gasparis AP, Kim PS, Dean SM, Khilnani NM, Labropoulos N (2020O) Diagnostic approach to lower limb edema. Phlebology 35(9):650–655 32631171 10.1177/0268355520938283 PMC7536506

[3] Mortimer PS. The pathophysiology of lymphedema. Cancer. 1998 Dec 15;83(12 Suppl American):2798–802. 10.1002/(sici)1097-0142(19981215)83:12b+<2798::aid-cncr28>3.3.co;2-5 9874400

[4] Lawenda BD, Mondry TE, Johnstone PA (2009J) Lymphedema: a primer on the identification and management of a chronic condition in oncologic treatment. CA Cancer J Clin 59(1):8–24 19147865 10.3322/caac.20001

[5] Dolmatova E, Waheed N, Olson BM, Patel SA, Mandawat A (2023J 1) The intersection of prostate cancer and hypertension: a call to action. Curr Treat Options Oncol 24(7):892–905 37191906 10.1007/s11864-023-01094-z

[6] Scott LJ (2017S) Abiraterone acetate: a review in metastatic castration-resistant prostate cancer. Drugs 77(14):1565–1576 28819727 10.1007/s40265-017-0799-9

[7] Zhu X, Wu S (2019J 1) Risk of hypertension in cancer patients treated with abiraterone: a meta-analysis. Clin Hypertens 25(1):12 31168403 10.1186/s40885-019-0116-x PMC6545007

[8] Lugtenberg RT, de Groot S, Houtsma D, Dezentjé VO, Vulink AJE, Fischer MJ et al (2023M 9) Phase 1 study to evaluate the safety of reducing the prophylactic dose of dexamethasone around docetaxel infusion in patients with prostate and breast cancer. Cancers 15(6):1691 36980577 10.3390/cancers15061691 PMC10046524

[9] Baker J, Ajani J, Scotté F, Winther D, Martin M, Aapro MS et al (2009F 1) Docetaxel-related side effects and their management. Eur J Oncol Nurs 13(1):49–59 19201649 10.1016/j.ejon.2008.10.003

[10] Schrijvers D, Wanders J, Dirix L, Prove A, Vonck I, van Oosterom A et al (1993A) Coping with toxicities of docetaxel (Taxotere). Ann Oncol Off J Eur Soc Med Oncol 4(7):610–611 10.1093/oxfordjournals.annonc.a058599 8103352

[11] Sudduth CL, Greene AK (2022M) Lymphedema and obesity. Cold Spring Harb Perspect Med 12(5) 35074795 10.1101/cshperspect.a041176 PMC9159261

[12] Abassi Z, Khoury EE, Karram T, Aronson D. Edema formation in congestive heart failure and the underlying mechanisms. Front Cardiovasc Med [Internet]. 2022 Sep 27 [cited 2024 Nov 3];9. Available from: https://www.frontiersin.org/journals/cardiovascularmedicine/articles/10.3389/fcvm.2022.933215/full 10.3389/fcvm.2022.933215 PMC9553007 36237903

[13] Eberhardt RT, Raffetto JD (2005M 10) Chronic venous insufficiency. Circulation 111(18):2398–2409 15883226 10.1161/01.CIR.0000164199.72440.08

[14] Epstein BJ, Roberts ME (2009D) Managing peripheral edema in patients with arterial hypertension. Am J Ther 16(6):543 19636244 10.1097/MJT.0b013e3181afbf9f

[15] Meiklejohn JA, Heesch KC, Janda M, Hayes SC. How people construct their experience of living with secondary lymphoedema in the context of their everyday lives in Australia. Support Care Cancer. 20120718th ed. 2013 Feb;21(2):459–66. 10.1007/s00520-012-1534-4 23010957

[16] Monteiro AJ, de Labra C, Losa-Iglesias ME, Dias A, Becerro-de-Bengoa-Vallejo R, Silva-Migueis H et al (2023J) Depressive symptoms and their severity in a sample with lymphedema: a case–control investigation. Front Psychiatry 5(14):1202940 10.3389/fpsyt.2023.1202940 PMC10354281 37476539

[17] Neuberger M, Schmidt L, Wessels F, Linke M, Müller C, Westhoff N et al (2022F) Onset and burden of lower limb lymphedema after radical prostatectomy: a cross-sectional study. Support Care Cancer Off J Multinatl Assoc Support Care Cancer 30(2):1303–1313 10.1007/s00520-021-06520-2 34477972

[18] DiSipio T, Rye S, Newman B, Hayes S. Incidence of unilateral arm lymphoedema after breast cancer: a systematic review and meta-analysis. Lancet Oncol. 20130327th ed. 2013 May;14(6):500–15. 10.1016/S1470-2045(13)70076-7 23540561

[19] Beesley V, Janda M, Eakin E, Obermair A, Battistutta D (2007J 15) Lymphedema after gynecological cancer treatment: prevalence, correlates, and supportive care needs. Cancer 109(12):2607–2614 17474128 10.1002/cncr.22684

[20] Ryan M, Stainton MC, Slaytor EK, Jaconelli C, Watts S, Mackenzie P (2003A) Aetiology and prevalence of lower limb lymphoedema following treatment for gynaecological cancer. Aust N Z J Obstet Gynaecol 43(2):148–151 14712972 10.1046/j.0004-8666.2003.00040.x

[21] Harris PA, Taylor R, Minor BL, Elliott V, Fernandez M, O’Neal L et al (2019J) The REDCap consortium: building an international community of software platform partners. J Biomed Inform 95 31078660 10.1016/j.jbi.2019.103208 PMC7254481

[22] Ward LC, Dylke E, Czerniec S, Isenring E, Kilbreath SL (2011M) Reference ranges for assessment of unilateral lymphedema in legs by bioelectrical impedance spectroscopy. Lymphat Res Biol 9(1):43–46 21417766 10.1089/lrb.2010.0024

[23] Froeding LP, Greimel E, Lanceley A, Oberguggenberger A, Schmalz C, Radisic VB et al (2018M) Assessing patient-reported quality of life outcomes in vulva cancer patients: a systematic literature review. Int J Gynecol Cancer 28(4):808–817 29420364 10.1097/IGC.0000000000001211

[24] Fu MR, Axelrod D, Cleland CM, Qiu Z, Guth AA, Kleinman R et al (2015O) Symptom report in detecting breast cancer-related lymphedema. Breast Cancer Targets Ther 15(7):345–352 10.2147/BCTT.S87854 PMC4621182 26527899

[25] Fu MR, Cleland CM, Guth AA, Kayal M, Haber J, Cartwright F et al (2013J) L-dex ratio in detecting breast cancer-related lymphedema: reliability, sensitivity, and specificity. Lymphology 46(2):85–96 24354107 PMC4040962

[26] Dylke ES, Schembri GP, Bailey DL, Bailey E, Ward LC, Refshauge K, et al. Diagnosis of upper limb lymphedema: development of an evidence-based approach. Acta Oncol. 20160622nd ed. 2016 Dec;55(12):1477–83. 10.1080/0284186X.2016.1191668 27333213

[27] Jensen K, Bonde Jensen A, Grau C. A cross sectional quality of life study of 116 recurrence free head and neck cancer patients. The first use of EORTC H&N35 in Danish. Acta Oncol. 2006 Jan 1;45(1):28–37. 10.1080/02841860500417536 16464793

[28] Aaronson NK, Ahmedzai S, Bergman B, Bullinger M, Cull A, Duez NJ et al (1993M 3) The European Organization for Research and Treatment of Cancer QLQ-C30: a quality-of-life instrument for use in international clinical trials in oncology. J Natl Cancer Inst 85(5):365–376 8433390 10.1093/jnci/85.5.365

[29] Chu D, Popovic M, Chow E, Cella D, Beaumont JL, Lam H et al (2014S) Development, characteristics and validity of the EORTC QLQ-PR25 and the FACT-P for assessment of quality of life in prostate cancer patients. J Comp Eff Res 3(5):523–531 25350803 10.2217/cer.14.41

[30] Olsen LR, Jensen DV, Noerholm V, Martiny K, Bech P (2003F) The internal and external validity of the Major Depression Inventory in measuring severity of depressive states. Psychol Med 33(2):351–356 12622314 10.1017/s0033291702006724

[31] Christensen KB, Packness A, Simonsen E, Brodersen J (2024J 1) Psychometric validation of the Danish version of the Major Depression Inventory using data from the Lolland-Falster health study (LOFUS). Nord J Psychiatry 78(5):392–401 38546419 10.1080/08039488.2024.2333445

[32] Noble-Jones R, Fitzpatrick B, Sneddon MC, Hendry DS, Leung HY (2014O) Development of the lymphoedema genito-urinary cancer questionnaire. Br J Nurs Mark Allen Publ 9(23 Suppl 18):S14-19 10.12968/bjon.2014.23.Sup18.S14 25302997

[33] Fokdal L, Berg M, Zedan AH, Mortensen B, Nissen HD, Bentzen L et al (2023O) Patient-reported lower limb edema after primary radiotherapy for prostate cancer. Acta Oncol Stockh Swed 62(10):1279–1285 10.1080/0284186X.2023.2251669 37647364

[34] Smith MR, Finkelstein JS, McGovern FJ, Zietman AL, Fallon MA, Schoenfeld DA et al (2002F 1) Changes in body composition during androgen deprivation therapy for prostate cancer. J Clin Endocrinol Metab 87(2):599–603 11836291 10.1210/jcem.87.2.8299

[35] Kao HH, Kao LT, Li IH, Pan KT, Shih JH, Chou YC et al (2019M) Androgen deprivation therapy use increases the risk of heart failure in patients with prostate cancer: a population-based cohort study. J Clin Pharmacol 59(3):335–343 30402905 10.1002/jcph.1332

[36] Bigaran A, Zopf E, Gardner J, La Gerche A, Murphy DG, Howden EJ et al (2021M) The effect of exercise training on cardiometabolic health in men with prostate cancer receiving androgen deprivation therapy: a systematic review and meta-analysis. Prostate Cancer Prostatic Dis 24(1):35–48 32860010 10.1038/s41391-020-00273-5

[37] Tiruye T, O’Callaghan M, FitzGerald LM, Moretti K, Jay A, Higgs B et al (2024A 1) Medication-based comorbidity measures and prostate cancer treatment selection. Clin Genitourin Cancer 22(2):599-609.e2 38369388 10.1016/j.clgc.2024.01.018

[38] Litwin MS, Tan HJ (2017J 27) The diagnosis and treatment of prostate cancer: a review. JAMA 317(24):2532–2542 28655021 10.1001/jama.2017.7248

[39] Facondo G, Bottero M, Goanta L, Farneti A, Faiella A, D’Urso P et al (2025M 18) Incidence and predictors of lower extremity lymphedema after postoperative radiotherapy for prostate cancer. Radiat Oncol 20(1):41 40102881 10.1186/s13014-025-02599-7 PMC11921733

[40] Kuehner C (2017F) Why is depression more common among women than among men? Lancet Psychiatry 4(2):146–158 27856392 10.1016/S2215-0366(16)30263-2

[41] Son A, O’Donnell TF, Izhakoff J, Gaebler JA, Niecko T, Iafrati MA (2019S) Lymphedema-associated comorbidities and treatment gap. J Vasc Surg Venous Lymphat Disord 7(5):724–730 31248833 10.1016/j.jvsv.2019.02.015

[42] Keast DH, Despatis M, Allen JO, Brassard A (2015J) Chronic oedema/lymphoedema: under-recognised and under-treated. Int Wound J 12(3):328–333 24618210 10.1111/iwj.12224 PMC7950664

[43] Shah C, Whitworth P, Valente S, Schwarz GS, Kruse M, Kohli M et al (2023F 1) Bioimpedance spectroscopy for breast cancer-related lymphedema assessment: clinical practice guidelines. Breast Cancer Res Treat 198(1):1–9 36566297 10.1007/s10549-022-06850-7 PMC9883343

[44] Russo S, Walker JL, Carlson JW, Carter J, Ward LC, Covens A et al (2020N 4) Standardization of lower extremity quantitative lymphedema measurements and associated patient-reported outcomes in gynecologic cancers. Gynecol Oncol 160(2):625 33158510 10.1016/j.ygyno.2020.10.026 PMC7946397

[45] Czerniec SA, Ward LC, Refshauge KM, Beith J, Lee MJ, York S et al (2010J 1) Assessment of breast cancer-related arm lymphedema—comparison of physical measurement methods and self-report. Cancer Invest 28(1):54–62 19916749 10.3109/07357900902918494

