# Supplementary figures and images for: Comprehensive assessment of lower limb edema and its association with quality of life among men with prostate cancer
Source: Support Care Cancer. 2025 Jun 16;33(7):586. doi: 10.1007/s00520-025-09613-4 (PMC12167717; doi:10.1007/s00520-025-09613-4)

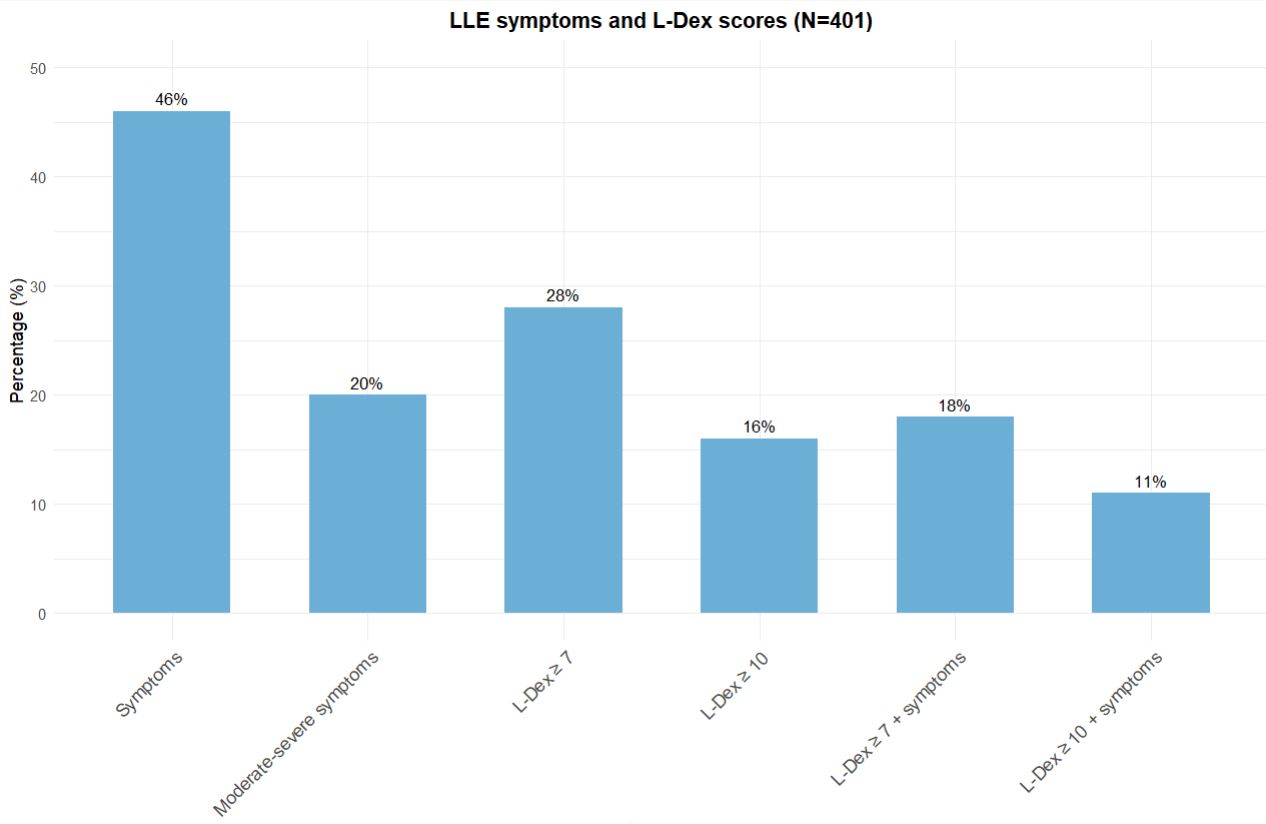

Supplement: Supplementary file 1 — (JPG 53.3 KB) [file 520_2025_9613_MOESM1_ESM.jpg]
