# Supplementary material for: Comprehensive assessment of lower limb edema and its association with quality of life among men with prostate cancer
Source: Support Care Cancer. 2025 Jun 16;33(7):586. doi: 10.1007/s00520-025-09613-4 (PMC12167717; doi:10.1007/s00520-025-09613-4)
Supplement: Supplementary file 2 — (DOCX 23.9 KB) [file 520_2025_9613_MOESM2_ESM.docx]

**Supplementary file 2. Stepwise backward selection using AIC**

|  | DF | Start | Step 1 | Step 2 | Step 3 | Step 4 | Step 5 | Step 6 | Step 7 | Step 8 | Step 9 | Step 10 | Step 11 | Step 12 | Step 13 | Step 14 | Step 15 |
| --- | --- | --- | --- | --- | --- | --- | --- | --- | --- | --- | --- | --- | --- | --- | --- | --- | --- |
|  |  | **AIC** | **AIC** | **AIC** | **AIC** | **AIC** | **AIC** | **AIC** | **AIC** | **AIC** | **AIC** | **AIC** | **AIC** | **AIC** | **AIC** | **AIC** | **AIC** |
| <none> | NA | 178.113 | 173.762 | 171.762 | 169.767 | 167.818 | 166.032 | 164.221 | 162.497 | 160.637 | 159.002 | 157.658 | 156.487 | 155.285 | 154.722 | 154.630 | 154.298 |
| Age | 1 | 177.159 | 173.278 | 171.300 | 169.355 | 167.392 | 165.497 | 163.902 | 162.276 | 160.412 | 159.334 | 158.360 | 157.912 | 157.538 | 157.163 | 156.232 | 155.636 |
| Heart failure | 1 | 186.408 | 181.414 | 179.508 | 177.530 | 175.882 | 174.345 | 172.590 | 170.991 | 170.296 | 169.472 | 167.516 | 166.349 | 164.930 | 164.178 | 163.636 | 155.636 |
| Valvular heart disease | 1 | 177.612 | 173.189 | 171.304 | 169.339 | 167.360 | 165.824 | 163.931 | 162.358 | 160.454 | 159.005 | 157.393 | 156.005 | 155.205 | 155.205 | 154.965 | 155.256 |
| Metabolic diseases | 1 | 179.684 | 175.077 | 173.081 | 171.140 | 169.411 | 167.765 | 166.371 | 164.633 | 162.682 | 160.868 | 159.687 | 158.384 | 157.825 | 157.556 | 157.088 | 156.988 |
| COPD or chronic bronchitis | 1 | 178.725 | 175.097 | 173.150 | 171.224 | 169.250 | 167.331 | 165.586 | 163.599 | 161.834 | 160.450 | 158.805 | 156.861 | 155.799 | 154.867 | 154.835 | 155.467 |
| BMI kg/m2 | 2 | 187.533 | 183.930 | 182.102 | 180.197 | 178.230 | 176.639 | 174.784 | 173.019 | 172.310 | 173.364 | 171.484 | 172.390 | 173.452 | 172.144 | 170.802 | 169.295 |
| Radiation therapy | 1 | 181.022 | 175.809 | 173.844 | 171.847 | 169.888 | 168.244 | 166.350 | 164.575 | 162.649 | 160.967 | 159.654 | 158.392 | 156.439 | 155.900 | 156.128 | 156.393 |
| Swelling before diagnosis | 2 | 190.562 | 186.693 | 185.055 | 183.169 | 181.202 | 179.206 | 177.564 | 176.008 | 175.735 | 174.929 | 173.229 | 171.309 | 169.445 | 171.072 | 169.620 | 167.624 |
| ADT | 1 | 178.566 | 176.542 | 174.606 | 172.617 | 170.633 | 168.761 | 166.972 | 165.347 | 163.521 | 161.997 | 160.970 | 160.273 | 158.760 | 158.321 | 158.081 | 157.750 |
| Diabetes | 1 | 178.009 | 173.512 | 171.512 | 169.550 | 167.582 | 165.956 | 164.055 | 162.574 | 160.942 | 159.224 | 157.956 | 157.128 | 155.731 | 155.062 | 154.298 |  |
| No. of LN extracted | 1 | 176.766 | 172.727 | 170.731 | 168.857 | 166.860 | 164.982 | 163.361 | 161.726 | 159.836 | 158.293 | 157.092 | 155.881 | 154.895 | 154.630 |  |  |
| Other cancers | 1 | 177.723 | 173.420 | 171.451 | 169.454 | 167.456 | 165.492 | 163.550 | 161.622 | 159.904 | 158.169 | 156.886 | 155.884 | 154.722 |  |  |  |
| Osteoarthritis | 1 | 177.033 | 172.755 | 170.758 | 168.761 | 166.861 | 164.961 | 162.977 | 161.390 | 159.715 | 157.992 | 156.642 | 155.285 |  |  |  |  |
| Crohn's disease | 1 | 176.893 | 172.596 | 170.596 | 168.643 | 166.652 | 164.916 | 163.197 | 161.444 | 159.585 | 157.890 | 156.487 |  |  |  |  |  |
| Other disease | 1 | 176.662 | 172.208 | 170.208 | 168.210 | 166.309 | 164.532 | 162.821 | 161.076 | 159.219 | 157.658 |  |  |  |  |  |  |
| Hypertension | 1 | 176.212 | 172.210 | 170.213 | 168.213 | 166.393 | 164.534 | 162.734 | 160.905 | 159.002 |  |  |  |  |  |  |  |
| Rheumatoid arthritis | 1 | 176.409 | 172.101 | 170.101 | 168.104 | 166.135 | 164.331 | 162.548 | 160.637 |  |  |  |  |  |  |  |  |
| Venous thrombosis | 1 | 176.437 | 172.167 | 170.167 | 168.171 | 166.196 | 164.303 | 162.497 |  |  |  |  |  |  |  |  |  |
| Chemotherapy | 1 | 176.580 | 171.992 | 169.996 | 168.003 | 166.047 | 164.221 |  |  |  |  |  |  |  |  |  |  |
| Osteoporosis | 1 | 176.181 | 171.989 | 169.992 | 168.007 | 166.032 |  |  |  |  |  |  |  |  |  |  |  |
| Elevated blood pressure | 1 | 176.237 | 171.807 | 169.808 | 167.818 |  |  |  |  |  |  |  |  |  |  |  |  |
| Prostatectomy | 1 | 176.233 | 171.766 | 169.767 |  |  |  |  |  |  |  |  |  |  |  |  |  |
| Blood clot | 1 | 176.124 | 171.762 |  |  |  |  |  |  |  |  |  |  |  |  |  |  |
| Stage | 3 | 173.762 |  |  |  |  |  |  |  |  |  |  |  |  |  |  |  |

**DF: Degrees of Freedom, AIC: Akaike Information Criterion, BMI: body mass index, ADT: androgen deprivation therapy, LN: lymph nodes, COPD: Chronic Obstructive Pulmonary Disease. The table shows the stepwise backward selection process using Akaike Information Criterion (AIC) to determine the best-fitting model. Starting with all candidate variables, variables were removed one at a time based on their impact on model fit, as measured by AIC. Lower AIC values indicate a better model. The process continued until no further improvements in AIC were possible, resulting in the final model with the lowest AIC value.**
