# Supplementary material for: Comprehensive assessment of lower limb edema and its association with quality of life among men with prostate cancer
Source: Support Care Cancer. 2025 Jun 16;33(7):586. doi: 10.1007/s00520-025-09613-4 (PMC12167717; doi:10.1007/s00520-025-09613-4)
Supplement: Supplementary file 3 — (DOCX 14.8 KB) [file 520_2025_9613_MOESM3_ESM.docx]

**Supplementary file 3. Prevalence of lower limb edema for specific treatments**

|  | All participants  N (%) | LLE  N (%) |
| --- | --- | --- |
| Radical prostatectomy  ∙ With PLND  ∙ Without PLND | 58 (15%)  58 (15%) | 2 (4%) 1 (2%) |
| Radical prostatectomy + ADT  ∙ With PLND  ∙ Without PLND | 25 (6%) 8 (2%) | 7 (28%) 0 (0%) |
| Radiation therapy + ADT | 38 (10%) | 5 (13%) |
| Chemotherapy + ADT | 25 (6%) | 2 (8%) |
| Radical prostatectomy + radiation therapy + ADT  ∙ With PLND  ∙ Without PLND | 23 (6%) 5 (1%) | 1 (4%) 0 (0%) |
| ADT | 39 (10%) | 10 (26%) |
| Radical prostatectomy + radiation therapy + chemotherapy + ADT  ∙ With PLND  ∙ Without PLND | 11 (3%) 1 (0.2%) | 3 (27%)  0 (0%) |

Prevalence is based on specific treatment combinations. For example, “Radiation therapy + Endocrine treatment” includes only patients receiving both treatments, but excludes those with additional treatments like chemotherapy or surgery. N: Number, PLND: pelvic lymph node dissection, ADT: androgen deprivation therapy.
