# Supplementary material for: Comprehensive assessment of lower limb edema and its association with quality of life among men with prostate cancer
Source: Support Care Cancer. 2025 Jun 16;33(7):586. doi: 10.1007/s00520-025-09613-4 (PMC12167717; doi:10.1007/s00520-025-09613-4)
Supplement: Supplementary file 4 — (DOCX 13.7 KB) [file 520_2025_9613_MOESM4_ESM.docx]

**Supplementary file 4. Depression measured by Major Depression Inventory**

|  | All N=401 | LLE N=45 | No LLE N=356 | P-value |
| --- | --- | --- | --- | --- |
|  | N (%) | N (%) | N (%) |  |
| Depression  ∙ No depression ∙ Mild depression ∙ Moderate depression ∙ Severe depression | 366 (91.3%)  9 (2.2%)  8 (2.0%) 16 (4.0%) | 40 (88.9%) 2 (4.4%) 1 (2.2%) 2 (4.4%) | 326 (92.1%) 7 (2.0%) 7 (2.0%) 14 (4.0%) | 0.763 |

The total score ranges from 0 to 50, with of ≤ 20 indicating no depression, 21-25 mild depression, 26-30 moderate depression, and ≥30 severe depression.
