# Supplementary material for: Comprehensive assessment of lower limb edema and its association with quality of life among men with prostate cancer
Source: Support Care Cancer. 2025 Jun 16;33(7):586. doi: 10.1007/s00520-025-09613-4 (PMC12167717; doi:10.1007/s00520-025-09613-4)
Supplement: Supplementary file 5 — (DOCX 15.5 KB) [file 520_2025_9613_MOESM5_ESM.docx]

**Supplementary file 5. Self-reported symptoms and impact of lower limb edema on daily activities measured by The Lymphoedema Genito-Urinary Cancer Questionnaire**

|  | Not at all  N (%) | A little  N (%) | Quite a bit  N (%) | Very much  N (%) |
| --- | --- | --- | --- | --- |
| The symptoms become more noticeable at end of day | 17 (39%) | 11 (25%) | 9 (21%) | 7 (16%) |
| Average severity of swelling this week | 10 (23%) | 21 (48%) | 7 (16%) | 6 (14%) |
| The symptoms are affecting: | | | | |
| Which clothes/shoes I can wear | 27 (60%) | 7 (16%) | 8 (18%) | 3 (7%) |
| My sitting | 40 (89%) | 3 (7%) | 2 (4%) | 0 (0%) |
| Getting in/out of bed | 38 (84%) | 4 (9%) | 3 (7%) | 0 (0%) |
| My walking | 26 (58%) | 11 (24%) | 4 (9%) | 4 (9%) |
| Passing urine | 37 (82%) | 7 (16%) | 0 (0%) | 1 (2%) |
| My sexual function | 39 (87%) | 1 (2%) | 0 (0%) | 5 (11%) |
| The skin around the swollen area: | | | | |
| Feels tight | 20 (46%) | 11 (25%) | 8 (18%) | 5 (11%) |
| Has changed color | 30 (68%) | 8 (18%) | 5 (11%) | 1 (2%) |
| Feels different | 22 (50%) | 11 (25%) | 8 (18%) | 3 (7%) |
| Feels wet/cold | 42 (96%) | 2 (5%) | 0 (0%) | 0 (0%) |
| The symptoms give me discomfort in: | | | | |
| In my leg(s) | 16 (36%) | 12 (27%) | 10 (23%) | 6 (14%) |
| In my genitals | 37 (84%) | 5 (11%) | 2 (5%) | 0 (0%) |
